# Structure and Properties of Silica Glass Densified in Cold Compression and Hot Compression

**DOI:** 10.1038/srep15343

**Published:** 2015-10-15

**Authors:** Michael Guerette, Michael R. Ackerson, Jay Thomas, Fenglin Yuan, E. Bruce Watson, David Walker, Liping Huang

**Affiliations:** 1 Department of Materials Science and Engineering, Rensselaer Polytechnic Institute, Troy, NY 12180, USA; 2 Department of Earth and Environmental Sciences, Rensselaer Polytechnic Institute, Troy, NY 12180, USA; 3 Department of Earth and Environmental Sciences, Lamont-Doherty Earth Observatory at Columbia University, Palisades, NY 10964, USA

## Abstract

Silica glass has been shown in numerous studies to possess significant capacity for permanent densification under pressure at different temperatures to form high density amorphous (HDA) silica. However, it is unknown to what extent the processes leading to irreversible densification of silica glass in cold-compression at room temperature and in hot-compression (e.g., near glass transition temperature) are common in nature. In this work, a hot-compression technique was used to quench silica glass from high temperature (1100 °C) and high pressure (up to 8 GPa) conditions, which leads to density increase of ~25% and Young’s modulus increase of ~71% relative to that of pristine silica glass at ambient conditions. Our experiments and molecular dynamics (MD) simulations provide solid evidences that the intermediate-range order of the hot-compressed HDA silica is distinct from that of the counterpart cold-compressed at room temperature. This explains the much higher thermal and mechanical stability of the former than the latter upon heating and compression as revealed in our *in-situ* Brillouin light scattering (BLS) experiments. Our studies demonstrate the limitation of the resulting density as a structural indicator of polyamorphism, and point out the importance of temperature during compression in order to fundamentally understand HDA silica.

As an archetypal network-forming oxide with rigid tetrahedral building blocks, silica glass (and melt) have been the targets of numerous high-pressure studies in condensed-matter physics, materials science and earth science, etc.^1^,^2^,^3^,^4^ Silica glass can undergo reversible and irreversible amorphous-amorphous (polyamorphic) transitions under pressure, leading to the elastic softening upon initial compression^5^,^6^,^7^,^8^,^9^ and permanent densification under high pressure^9^,^10^,^11^,^12^,^13^,^14^,^15^. At room temperature (cold-compression), at pressures above 8–9 GPa, irreversible polyamorphic transition takes place and the recovered glass has an increased density, reaching a maximum densification of ~21% after compression at 18–20 GPa^6^,^9^,^15^,^16^. The same or even higher amount of densification can be achieved under much lower pressures (4–8 GPa) at high temperatures (hot-compression)^13^,^17^,^18^,^19^,^20^,^21^,^22^,^23^,^24^. Till now, it remains elusive whether the same structural transformation takes place during the cold-compression and the hot-compression of silica glass, and whether the structure and properties of HDA silica can be understood solely based on the resulting density.

In this study, we compressed samples of silica glass to pressures up to 8 GPa, held them at 1100 °C (T_g_ ≈ 1200 °C) for 30 minutes, and rapidly quenched them to room temperature before releasing the pressure. At ambient conditions, the structure and properties of HDA silica obtained in the hot-compression herein were found to be distinct from those of the cold-compressed one in both experimental characterizations such as X-ray diffraction, Raman and BLS, and in MD simulations. *In-situ* BLS studies under high temperature and high pressure provide solid evidences that the thermal and mechanical stability of hot-compressed and cold-compressed HDA silica are very different. Our MD simulations reveal the different atomic processes involved in the cold-compression and the hot-compression of silica glass, and the structural differences in HDA silica obtained from different routes. Our study shows the limitation of the resulting density as a structural indicator of polyamorphism and the importance of temperature during compression in order to fundamentally understand the polyamorphic transitions in silica glass.

## Results

**Figure 1:**
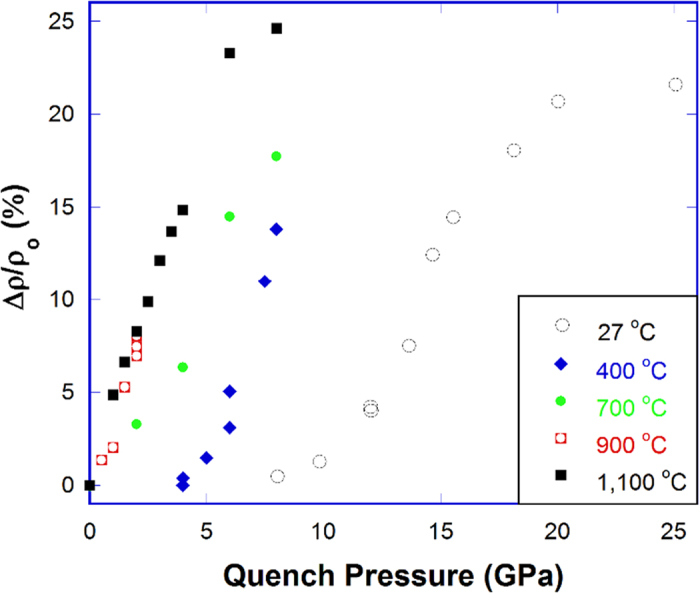
Density increase of silica glass as a function of quench pressure at different temperatures. Data at 27 °C were taken from Rouxel *et al.*^15^ and Deschamps *et al.*^12^, at 400 °C from Mackenzie^13^ and Arndt and Stöffler^17^, at 700 °C from Poe *et al.*^18^, at 900 °C from Hofler and Seifert^19^ by the authors and plotted together with our data at 1,100 °C.

Densification of silica glass as a function of quench pressure from the hot-compression in this study is shown in Fig. 1, together with data from the cold-compression at room temperature done by Rouxel *et al.*^15^ and Deschamps *et al.*^12^, at 400 °C by Mackenzie^13^ and Arndt and Stöffler^17^, at 700 °C by Poe *et al.*^18^, at 900 °C by Hofler and Seifert^19^. Figure 1 clearly shows that the amount of permanent densification depends on the temperature at which the compression was carried out. The difference becomes smaller as the temperature approaches the T_g_ of pristine silica glass. For example, the hot-compression at 900 °C in Hofler and Seifert’s study^19^ and at 1100 °C in this work give almost the same amount of densification. Figure 1 also shows that the hot-compression in the non-rigid state (near T_g_ of pristine silica glass) is much more effective than the cold-compression in the rigid state at room temperature in increasing the density of silica glass. Under 8 GPa quench pressure, the density of silica glass increases by nearly 25% in our study, compared to ~21% maximum increase achieved by the cold-compression under much higher pressures at 18–20 GPa^6^,^9^,^15^,^16^. The above observations show that temperature facilitates the densification of silica glass under pressure, probably by enabling different structural transformations otherwise not possible at room temperature.

**Figure 2:**
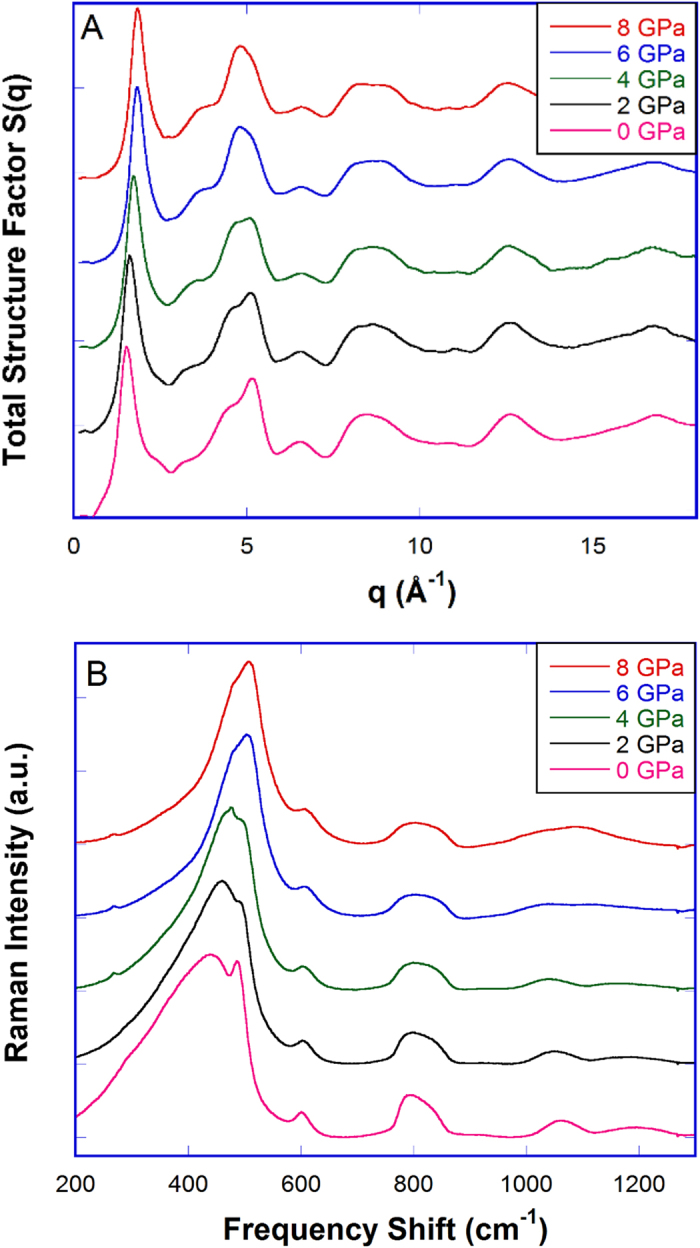
Structure of silica glass hot-compressed under 2, 4, 6 and 8 GPa at 1100 °C, compared to that of pristine silica glass (0 GPa). (**A**) Total structure factor S(q) and (**B**) Raman spectra. All measurements were carried out at ambient conditions. Spectra of samples quenched under 2 to 8 GPa are shifted vertically for clarity.

Total X-ray structure factor S(q) of hot-compressed samples at ambient conditions are shown in Fig. 2A. The position of the first sharp diffraction peak (FSDP), and its full width at half maximum (FWHM) can be seen in Fig. S4. The FSDP is related to the intermediate-range order of silica network with a correlation length (R): 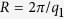, where 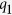 is the position of the FSDP. The coherence length (L) estimates the range over which the periodicity survives, and can be calculated as: 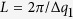, where 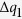 is the FWHM of the FSDP^25^. As in the case of densified silica glass in cold-compression, the position of the FSDP of hot-compressed silica shifts to higher *q* values with the increase of quench pressure, resulted from a compacted structure. However, contrary to cold-compression^26^, the FSDP of hot-compressed silica narrows (Fig. S4B) and its intensity does not decrease appreciably with pressure as seen in Fig. 2A, similar to the observations from our MD simulations in Fig. S5. These behaviors have also been observed by *in-situ* measurements of compressed silica glass being simultaneously heated in Inamura *et al.*’s study^21^ or in HDA silica densified at 500 °C^27^. This indicates that the intermediate-range order of cold-compressed silica is substantially altered under pressure. Broadening of the FSDP indicates that the coherence length dramatically decreases, or the intermediate-range order of cold-compressed silica glass becomes heterogeneous. On the contrary, narrowing of the FSDP in hot-compressed HDA silica seen in Fig. S4B shows that the responsible structural features in the intermediate-range are less varied with the increase of quench pressure. The coherence length is 10.12 Å for pristine glass silica glass, and 11.55 Å for the HC-8 GPa sample (quenched from 8 GPa at 1100 °C), a 12.4% increase. Discussion of the real-space correlation function G(r) can be found in the Supporting Information Sec. 5.

The effect of quench pressure on the structure of silica glass can be also seen from the Raman spectra at ambient conditions in Fig. 2B. The Raman main band (~440 cm^−1^) gradually narrows and shifts to 495 cm^−1^ with the increase of quench pressure from 0 to 8 GPa (Fig. S7A). This indicates a smaller mean value and a narrower distribution of Si-O-Si angles in hot-compressed silica glass. For comparison, the Raman main band has been shown to shift from 440 cm^−1^ to 530 cm^−1^ in cold-compressed silica glass within the elastic recovery regime of P < 8–9 GPa^9^. In our hot-compressed samples, these structural changes were frozen in during the quench process and became permanent at ambient conditions. The sharp D_1_ (~490 cm^−1^) and D_2_ (~605 cm^−1^) peak are attributed to breathing mode vibrations of 4- and 3-membered rings of Si and O^28^,^29^. Shifts of D_1_ and D_2_ peak to higher frequencies are hypothesized to be caused by slight buckling of these rings with densification^30^. A pronounced shift to lower frequency of the weak high-frequency bands (1060 cm^−1^ and 1200 cm^−1^) associated with Si–O stretching motions is observed in Fig. S7B, indicating a lengthening of Si–O bonds with increasing densification as seen in Fig. S6B. Raman spectra of cold-compressed and hot-compressed silica glass with a similar densification (~14% density increase) are shown in Fig. S8. The higher D_2_ intensity is usually attributed to the increase of the concentration of 3-membered rings in compacted glass^28^,^31^. But it may also mean that the network to which 3-membered rings are bonded and in which they vibrate is substantially modified during the cold-compression process.

**Figure 3:**
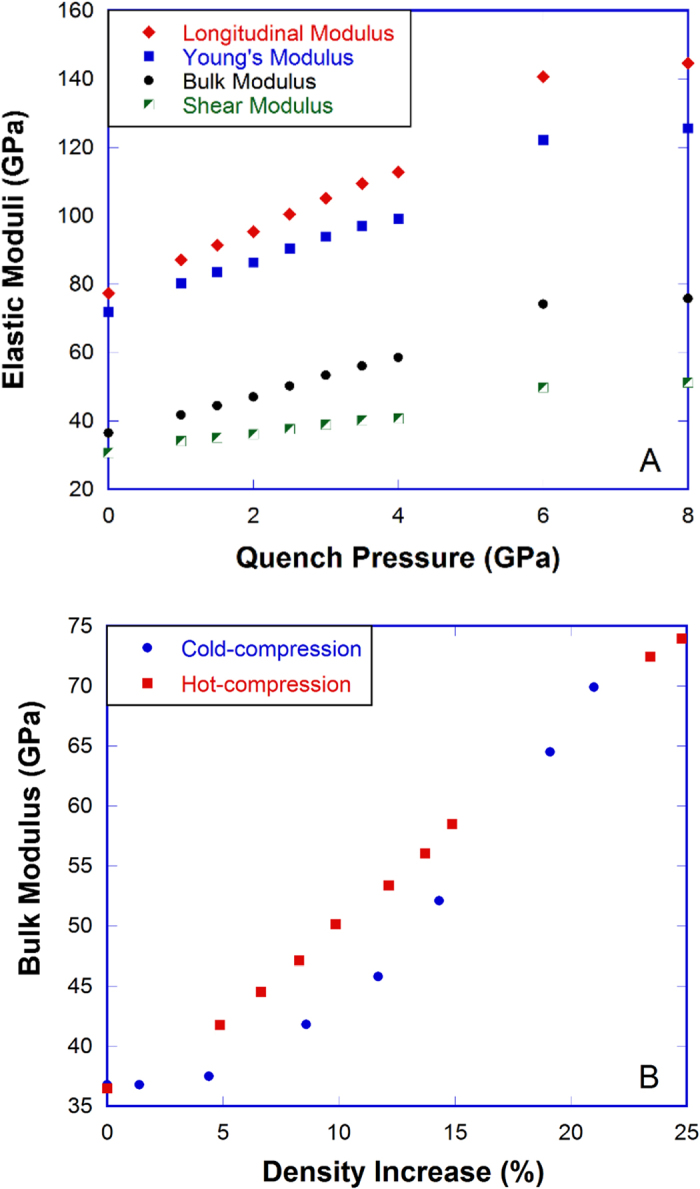
Elastic moduli of densified silica glass. (**A**) Elastic moduli of hot-compressed silica glass as a function of quench pressure. (**B**) Bulk modulus vs. density increase of silica glass from the cold-compression at room temperature^12^,^15^ and the hot-compression at 1100 °C in this study. Note: elastic moduli were all measured at ambient conditions.

Changes in the structure of silica glass as a function of quench pressure in the hot-compression lead to a substantial increase of elastic moduli as seen in Fig. 3A (details on elastic moduli measurements can be found in the Supporting Information Sec. 7). When the quench pressure is increased from 0 to 8 GPa, the Young’s modulus increases from 72 to 123 GPa (71%), and shear modulus increases from 32 to 50 GPa (61%), much higher than the density increase (25%) in Fig. 1. Figure 3B shows that below 20% density increase, HDA silica from the hot-compression has a higher bulk modulus than that from the cold-compression for the same amount of densification. Above 20% density increase, the difference becomes smaller with the increase of densification. Similar trends can be seen in the Young’s modulus and shear modulus in Fig. S10. Contrary to the conclusion drawn by Deschamps *et al.*^12^, our study shows that elastic moduli depend not only on the amount of densification, but also on the pathway to reach the permanent densification. The above observations show that there is some difference between cold-compressed and hot-compressed HDA silica that is not distinguished by density.

**Figure 4:**
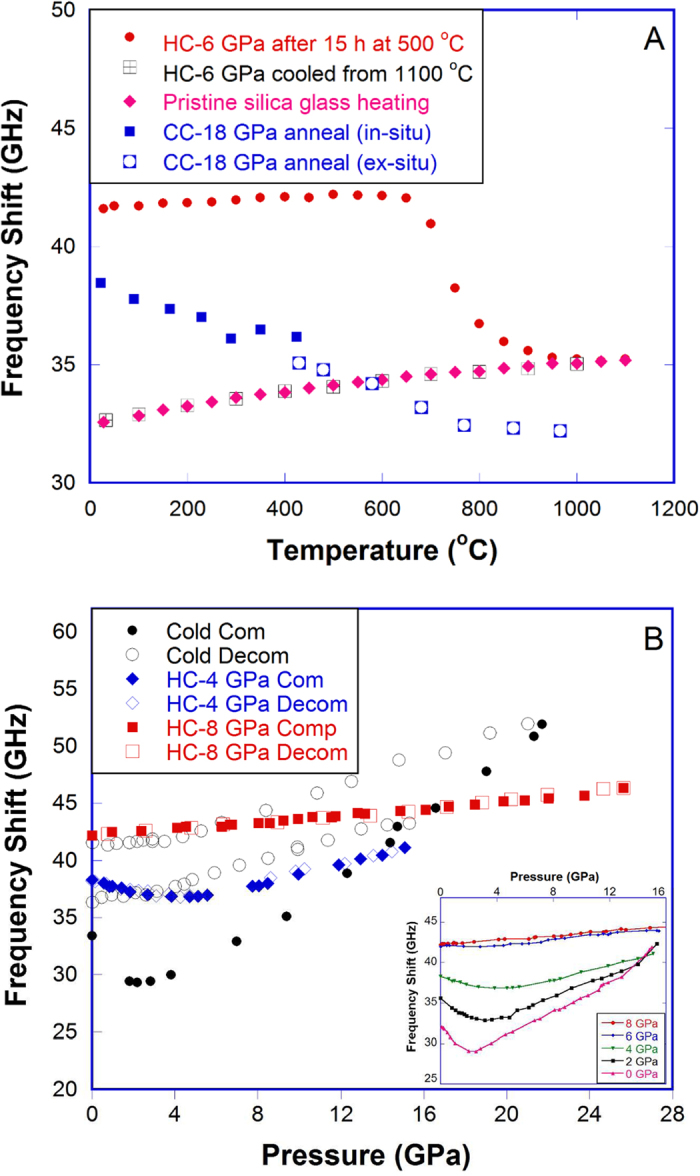
Thermal and mechanical reponse of densified silica glass. Longitudinal Brillouin frequency shift as a function of temperature (**A**) and pressure (**B**) for cold-compressed and hot-compressed silica glass. Note: Data for cold-compressed silica glass in (**A**) was taken from Grimsditch^32^, where the closed squares were measured at the specified temperature (*in-situ*) and the open squares were measured at room temperature after annealing at the specified temperature (*ex-situ*), each after an hour annealing. Data in this study were all measured *in-situ,* after 10 minutes at the specified temperature. Data for cold-compression of silica glass in (**B**) was taken from Sonneville *et al.*^9^. Inset: longitudinal Brillouin frequency shift of hot-compressed silica glass as a function of testing pressure during compression up to 15–16 GPa at room temperature.

Cold-compressed and hot-compressed HDA silica are truly set apart from one another by their dramatically different response to thermal and mechanical agitations. As seen in Fig. 4A, for the HC-6 GPa sample (quenched from 6 GPa at 1100 °C, 23% density increase), after being held at 500 °C for 15 h, there is negligible change in the longitudinal Brillouin frequency shift (from which the longitudinal modulus can be calculated given the sample density and refractive index). When temperature is higher than 650 °C, the Brillouin frequency shift decreases quickly and joins that of pristine silica glass at temperatures above 1000 °C. After that, there is no difference between the HC-6 GPa sample and pristine silica glass, as the cooling curve of the former overlaps perfectly with the heating curve of the latter. While for the CC-18 GPa sample (quenched from 18 GPa at room temperature, 17% density increase), the Brillouin frequency shift decrease starts even at room temperature^32^. The dramatic differences in the response to temperature indicates that the structure of the hot-compressed and cold-compressed HDA silica are unequivocally distinct.

As seen in Fig. 4B and in the inset, for pristine silica glass cold-compressed at room temperature, the Brillouin frequency shift initially decreases with pressure, reaches a minimum around 2–3 GPa, then increases as a normal solid. This is related to the well-known elastic anomaly of silica glass under pressure^5^,^6^,^7^,^8^,^9^. With the increase of quench pressure, the minimum in the frequency shift decreases and displaces to higher testing pressure, and eventually disappears in the HC-6 and HC-8 GPa samples as seen in the inset of Fig. 4B. For the HC-6 GPa sample, the frequency shift does not change with pressure up to 5–6 GPa, then adopts a slightly positive slope similar to that of the HC-8 GPa sample. Our study is in good agreement with previous studies^6^,^9^,^33^,^34^ and with observations from our MD simulations (Fig. S12) that the elastic anomaly progressively vanishes with the degree of densification. As shown in Fig. 4B, larger pressures (P > 15 GPa) are needed in the cold-compression in order to eliminate the elastic anomaly. Grimsditch showed that the cold-compression beyond the elastic deformation regime leads to densified silica glass that is then capable of elastic deformation upon re-compression to the maximum pressure of the initial compression^6^. Therefore, the re-compression curve of the CC-15 GPa and CC-22 GPa samples in Fig. 4B would overlap with the decompression curves from 15 GPa and 22 GPa^9^, which provides us an opportunity to compare the response of cold-compressed and hot-compressed silica glass to testing pressure. Figure 4B shows that the structural alterations of silica glass by the cold-compression is remarkably different from the hot-compression: 1) much higher pressures are required to reach comparable Brillouin frequency shifts (15 GPa and 22 GPa in the cold-compression versus 4 and 8 GPa in the hot-compression); 2) there is no elastic minimum in the CC-15 GPa sample, while a pronounced minimum still exists in the HC-4 GPa sample; 3) the CC-22 GPa sample shows much higher increase in the Brillouin frequency shift with the increase of testing pressure, compared with the HC-8 GPa sample. Moreover, the HC-6 GPa and HC-8 GPa samples remain elastic up to 20 GPa and 26 GPa (Fig. S11), respectively, which are the maximum pressure tested in each case. This shows that hot-compressed samples have a much higher threshold for irreversible structural changes, up to three times that of pristine silica glass that can be irreversibly densified at 8–9 GPa^6^,^9^,^15^,^16^.

## Discussion

The above observations show that the hot-compression in the non-rigid state can lead to unique structure and properties that cannot be achieved by the cold-compression in the rigid state. In contrast to conclusions drawn from previous studies of hot-compressed and cold-compressed HDA silica at ambient conditions^12^,^24^, our *in-situ* BLS measurements of the response of HDA silica to thermal and mechanical agitations show that the structure and properties of HDA silica strongly depend on the temperature at which the compression is carried out, not just the resulting densification.

**Figure 5:**
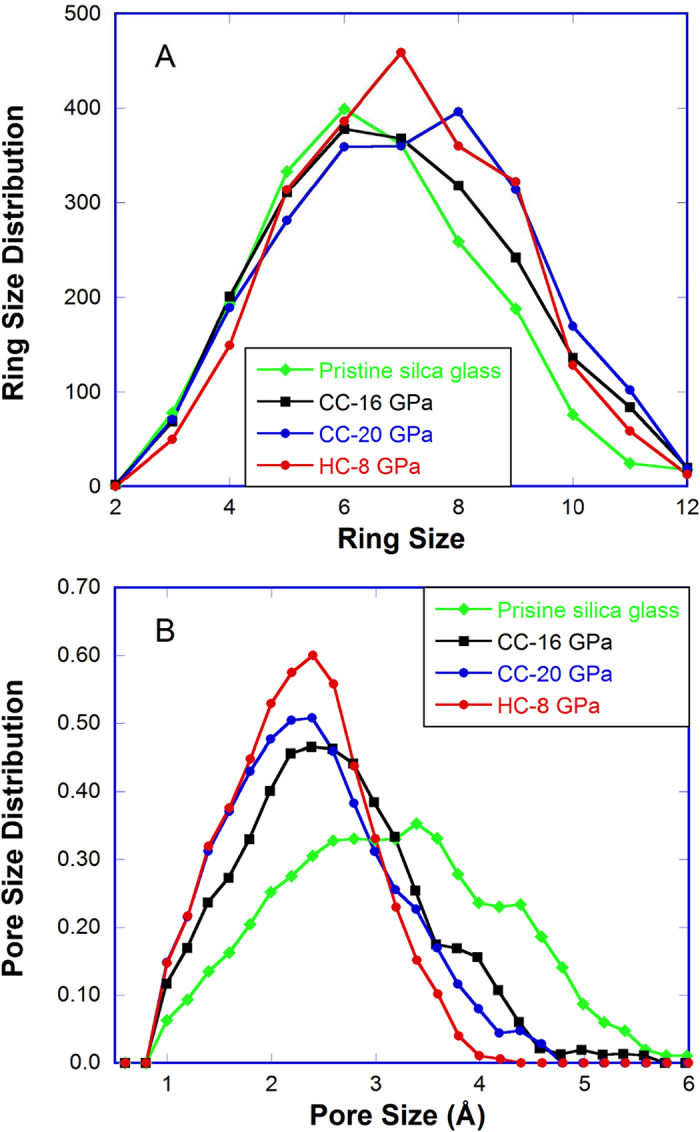
Structure of densified silica glass from MD simulations. (**A**) Ring size disirbution and (**B**) pore size distribution in pristine, cold-compressed and hot-compressed silica glasses from MD simulations.

The unique response of hot-compressed HDA silica to thermal and mechanical agitations in Fig. 4 no doubt results from the distinct underlying structure as seen in Fig. 2, although atomic scale details are hard to obtain from experiments alone. To this end, we carried out classical MD simulations to illustrate the atomic processes involved in the compression in the rigid and not-rigid state and the structural differences between the cold-compressed and hot-compressed HDA silica. Figure 5A shows the ring statistics of pristine, cold- and hot-compressed silica glass. Similar to previous first principles^35^ and classical MD simulations^36^, six-membered rings are the most abundant over a broad range distribution of rings in pristine silica glass. With the increase of densification in the cold-compression, the population of six-membered rings decreases, more and more larger rings (eight and higher) appear. In other words, the ring size distribution is stretched and skewed towards the right. In the HC-8 GPa sample, seven-membered rings become dominant, but the ring-size distribution is more or less symmetric, similar to that in pristine silica glass. This shows that the intermediate-order (or network connectivity) in cold-compressed and hot-compressed HDA silica are different. Further evidence is seen in the pore size distributions in Fig. 5B. In pristine silica glass, a broad range of pores (1–6 Å in diameter) exist. With the increase of densification in cold-compressed silica, the size of the most probable pores reduces, corresponding to a compacted structure. However, a substantial proportion of pores with diameters of 4–5 Å is still present. In hot-compressed silica, on the other hand, these large pores are completely eliminated, and the pore size distribution becomes narrower and more symmetric. Figure 5B clearly shows that the intermediate-range order in hot-compressed HDA silica is more uniform (homogeneous) than that in the cold-compressed counterpart. This is consistent with XRD measurements that a narrower and more intense FSDP is observed in the former than in the latter. Figure 5A also shows that in cold-compressed HDA silica, the population of 3- and 4-membered rings do not change appreciably relative to pristine silica glass. The higher intensity of the D_2_ peak in the Raman spectrum of cold-compressed silica glass in Fig. S8 more likely results from the substantial re-arrangements in the network structure to which these small rings are bonded and in which they vibrate.

The inhomogeneous intermediate-range order in cold-compressed HDA silica is inherited from the starting material. As pristine silica glass is an inhomogeneous solid at the atomic scale; upon compression in the rigid state, different regions respond to pressure differently. Certain regions are deformed more than others, leading to larger structural re-arrangements locally. Upon decompression, part of these structural modifications are quenched to ambient conditions, naturally leading to an inhomogeneous, but denser solid. On the other hand, decrease of viscosity, hence decrease of T_g_ with pressure for silica glass is expected from MD simulations^37^ and from viscosity measurements for materials with similar structures, such as water, silicate and germania liquids at high pressures^38^,^39^,^40^. Upon compression in the non-rigid state, due to the short relaxation times for viscous flow, a homogeneous denser equilibrium liquid structure can be quickly attained through coordinated tetrahedral movements and then frozen in at the glass transition temperature upon quenching, giving rise to a homogeneous denser glass. Substantial structural arrangements are needed in both cold- and hot-compressed HDA silica in order to accommodate the compacted packing of SiO_4_ tetrahedra. However, cold-compressed HDA silica has the structure of a dense and dis-organized glass compacted in the rigid state, while the hot-compressed counterpart has the structure of a dense frozen and well-organized liquid compacted in the non-rigid state. In both the cold-compression and the hot-compression, transient 5- and 6-fold Si species may form under pressure due to the displacive rather than thermally activated mechanism associated with the coordination change^41^,^42^. However, upon the release of pressure, these higher coordination states of Si revert back to the four-fold coordination state^26^,^43^,^44^,^45^. Based on a recent study of the oxygen number density^46^, even our hot-compressed HDA silica of highest density increase (25%) is still within the expected range for a fully polymerized tetrahedral framework.

The different intermediate-range order of cold- and hot-compressed HDA silica explains their different response to temperature and pressure. In cold-compressed HDA silica, an appreciable amount of large pores (>4 Å in diameter) still exist, which provide the open space (free volume) needed for structural re-arrangements upon annealing. Without them, the compacted structure from the hot-compression can be retained at much higher temperatures before reverting back to the non-densified state, thus a much higher thermal stability compared to the cold-compressed one (Fig. 4A). The heterogeneous intermediate-range structure of cold-compressed HDA silica also makes it prone to further structural re-arrangements, such as the coordination change under pressure, which explains the faster increase in the Brillouin frequency shift with pressure than that in the hot-compressed counterpart (Fig. 4B).

## Methods

The starting silica glass used is Suprasil 300 with low OH content (≤1 ppm). Samples quenched under pressures up to 4 GPa were prepared in a piston-cylinder (PC) apparatus^47^, while under 4 to 8 GPa were carried out in a Walker-type multi-anvil (MA) device^48^,^49^. Cylinders of 3 mm in diameter were core-drilled from a puck of the starting material and cut to 6 mm in length for PC experiments and 3 mm in length for use in MA. Details of sample preparation, and differences in applied pressures between the two apparatus are discussed in Supporting Information Sec. 1 & *2*.

A Metricon Model 2010/M Prism Coupler was used to measure the room temperature refractive index (accuracy of ±0.0002) with a 532 nm green laser. Density of our hot-compressed samples were determined from the linear refractive index vs. density relationship established by Tan *et al.*^50^ and shown in Fig. S3.

X-ray diffraction at ambient conditions was measured at beamline X17B3 at the National Synchrotron Light Source (NSLS) at the Brookhaven National Lab, operated through COMPRES. Angle dispersive scattering was carried out by using monochromatic X-ray with wavelength of 0.152901 ± 0.0001 Å^51^, focused to ~15 μm at distance of L = 293.73 mm from a CCD detector with an area of 1,678 cm^2^. The distance and energy was calibrated by CeO_2_ powder diffraction using an iterative ring matching method^51^. This enabled radially symmetric study of *q* values to 24 Å^−1^, but due to noise at higher *q* range, the spectra were truncated at 18 Å^−1^. The Fit2D program^52^ was used to process raw X-ray diffraction data to generate 1-dimensional intensity vs. 2*θ* plots, and the RAD program^53^ was used to generate S(q) and G(r) spectra.

A six-pass high contrast Fabry-Pérot interferometer coupled with a LabRAM HR800 confocal Raman microscope were used to carry out light scattering experiments by using a 532 nm green laser as the probing light source. Raman scattering was done in backscattering (180°) geometry with a 50× microscope lens and a 600 gr/mm grating. An emulated platelet geometry was used in BLS measurements by placing the sample on a well-polished Pt plate^54^. *In-situ* BLS during annealing was carried out by using a Linkam TS1500 heating stage. The sample was heated at a rate of 50 °C/min to the target temperature (±1 °C) and allowed to equilibrate for 10 minutes before Brillouin spectra were taken for ~2–5 minutes. A membrane-driven DAC was used to generate hydrostatic pressures up to 26 GPa. A glass sample (~100 μm × 100 μm × 20 μm in size), ruby ball (5–10 μm in diameter, as pressure calibrant) and pressure transmitting medium (PTM) were loaded into a hole drilled in a stainless steel gasket. Pressure in the DAC was determined from the pressure dependent ruby fluorescence shift^55^. Hydrostatic conditions up to 15 GPa were achieved by using 4:1 methanol: ethanol mixture as the PTM. For tests above 15 GPa, liquid Argon was cryogenically loaded into DAC to act as the PTM. After certain pressure was reached in the DAC, the sample was allowed to equilibrate for 15–20 minutes before any measurement was taken. Longitudinal frequency shift was obtained from the BLS backscattering geometry in the DAC by using a lens with f = 50.8 mm. Repeated measurements gave errors in Brillouin frequency shifts to be ~0.1 GHz, errors in pressures were estimated by repeated measurements to be within 0.1 GPa.

Molecular dynamics (MD) simulations were carried out for 3000 particles (1000 Si and 2000 O) with periodic boundary conditions, using a charge-transfer three-body potential^56^. The hot-compressed samples were obtained by heating and melting cristobalite silica and subsequently quenching the liquid with different pressures on the simulation box^34^. The pressures were released and later tests were all done at room temperature. For the cold-compressed samples, to overcome the limitation of time in MD simulation, a pristine silica glass was heated to 727 °C (T_g_ at ~2700 °C in MD) and compressed to different pressures to facilitate the densification process. Pressures were released at 727 °C and samples were cooled down to room temperature for further tests. Density of HC-8 GPa sample from our MD simulation is 2.750 g/cc, close to the value of 2.742 g/cc for the sample hot compressed under the same pressure in our experiment. More details on the sample preparation and the ring size distribution analysis can be found in our previous studies^34^,^36^,^57^,^58^. Pore size distribution was calculated by following the procedures in Gelb and Gubbins’ work^59^.

## Additional Information

**How to cite this article**: Guerette, M. *et al.* Structure and Properties of Silica Glass Densified in Cold Compression and Hot Compression. *Sci. Rep.* **5**, 15343; doi: 10.1038/srep15343 (2015).

## Supplementary Material

Supporting Information
