# Supplementary material for: Structure and Properties of Silica Glass Densified in Cold Compression and Hot Compression
Source: Sci Rep. 2015 Oct 15;5:15343. doi: 10.1038/srep15343 (PMC4606793; doi:10.1038/srep15343)
Supplement: Supporting Information [file srep15343-s1.pdf]

Supporting Information for "Structure and Properties of Silica Glass Densified in Cold Compression and Hot Compression"

Michael Guerette[1], Michael R. Ackerson[2], Jay Thomas[2], Fenglin Yuan[1], E. Bruce Watson[2], David Walker[3] and Liping Huang[1,*]

[1]Department of Materials Science and Engineering, Rensselaer Polytechnic Institute, Troy, NY 12180, USA

[2]Department of Earth and Environmental Sciences, Rensselaer Polytechnic Institute, Troy, NY 12180, USA

[3]Department of Earth and Environmental Sciences, Lamont-Doherty Earth Observatory at Columbia University, Palisades, NY 10964, USA

1. Synthesis details.

Schematics of the sample cell assembly for hot-compression in piston-cylinder (PC) and multi-anvil (MA) experiments can be seen in Fig. S1. 19-mm diameter pressure assemblies were used for 1 and 1.5 GPa PC experiments, and 13-mm diameter pressure assemblies were used for 2-4 GPa PC experiments. PC cell assemblies consist of NaCl outer sleeve, a Pyrex glass thermal insulator sleeve, and a tubular high purity graphite heater with MgO filler pieces inside the heater which axially adjoin a centered sample (±0.1 mm). Samples quenched under pressures of 1-1.5 GPa were insulated from the heater by a MgO sleeve, samples under 2-3 GPa were not insulated from the heater, and those under 3.5-4 GPa were insulated from the heater by a thin fired pyrophyllite sleeve. Selected sleeve material or lack thereof was determined to have a negligible effect for PC experiments. Temperature was monitored with an Al_2O_3-insulated $W_{97}Re_3$-$W_{75}Re_{25}$ thermocouple terminated against a 1 mm thick MgO disc above the centered glass sample. Heating to 1100°C caused Pyrex to soften and decrease of the NaCl resistance to shear flow, allowing the cell material to deliver near-hydrostatic pressure inside the cylinder bore from axial pressure delivery of the piston. Pressure was overshot by 10-20% to allow the cell pressure to decay to the target value, requiring only minor upward adjustments to piston pressure during the first several minutes at 1100°C. Error in applied pressure has been previously estimated as ~20 MPa[1] for the PC apparatus.

1-inch tungsten carbide cubes with truncated edge lengths (TEL) of 8 mm were used for 4 and 6 GPa experiments in the MA apparatus, and 6 mm TEL cubes were used for the 8 GPa

* Correspondence and requests for materials should be addressed to Liping Huang (huangL5@rpi.edu)

experiment. Samples were centered between MgO filler pieces (±0.1mm) within a bored octahedron of 6 mm or 8 mm edge triangular faces to match the TEL cube faces, made with castable ceramic Aremco 584OF with integral stabilization fins (Fig. S1B). $LaCrO_3$ was used as heaters in MA experiments without insulation or barrier between the sample and the heater, and $LaCrO_3$ end caps were used as electric leads for heating. Temperature was monitored with a Pt-Pt_{90}/Rh_{10} (type S) thermocouple junction imbedded within MgO powder at the base of our centered sample, the leads of which were encased in Mullite sleeves and passed through holes drilled in the fins of the ceramic octahedron to then pass between the tungsten carbide cubes. No overshoot strategy was necessary with the MA device. Calibrations on the coesite to stishovite phase transition at 1200°C were used to deduce the pressure for the force applied, giving 5% error in applied pressure for the MA device[2]. Force of 240, 350 and 250 tons were applied in the 4, 6 and 8 GPa experiments, respectively.

The expected temperature variation for each sample shown is within ±25°C around the target temperature of 1100°C for both pressure devices[1-3]. Our synthesis temperature was chosen to allow the glass to soften but not enough structural mobility to aid crystallization, and our hold time of 30 minutes was estimated to be long enough to allow the desired structural reorganization but not enough for crystallization. In previous molecular dynamics (MD) simulations that inspired this study, silica melt was quenched under various pressures to form densified glass without crystallization due to the very fast quench rates used[4]. For the PC apparatus, circulating water above and below the thermally massive pressure vessel cooled the sample from 1100°C to <300°C in ~15-20 s. At these laboratory timescales, previous work showed that silica glass quenched from 1-3 GPa after being held at 1200°C for 60 minutes in the PC device had a tendency to crystallize [5]. In this study, there was no crystallization issue when quenching from 1100°C in the pressure range of 1-4 GPa with the PC device, though at pressures of 4-8 GPa in the MA device we had peripheral crystallization of coesite as determined by Raman spectroscopy, though completely amorphous internal regions remained. Increased crystallization at higher pressures indicate an apparent reduction in T_g with pressure, though significant differences exist between the MA and PC devices. Even though the MA device was not water cooled, the smaller thermal mass of the sample assembly and thermally conductive WC cubes and ceramic pressure medium allow for cooling from 1100°C to <300°C in ~2 s. The depressurization routine of the MA apparatus was done over 6-10 hours to retain stability of the WC cubes, rather than several minutes for depressurization of the PC apparatus.

Retrieved samples showed dilatational fractures upon cold, uniaxial unloading from the PC apparatus, yielding several discs of ~0.6 mm in thickness. MA samples remained continuous but were sectioned for analysis.

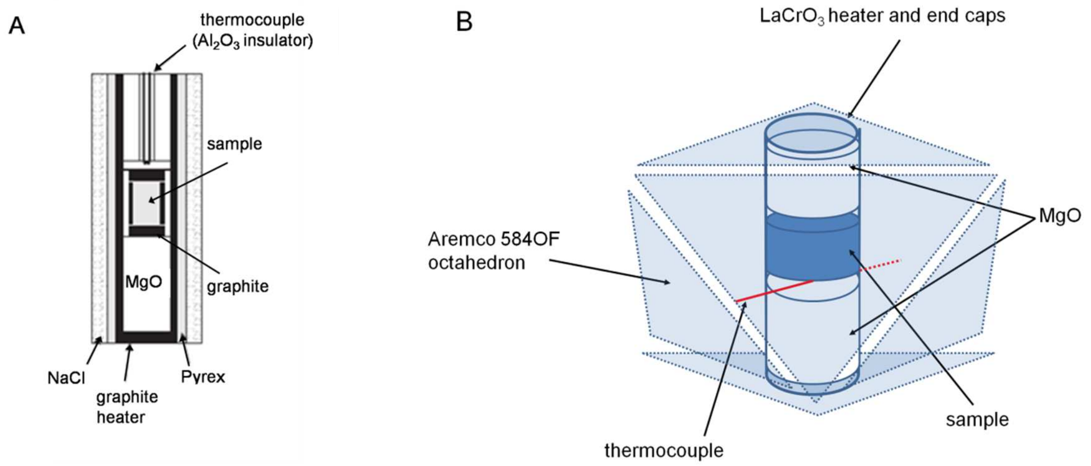

Fig. S1. Schematic of the sample cell assembly for hot-compression of silica glass (A) in a piston-cylinder (PC) apparatus in Dr. E.B. Watson's lab at Rensselaer, and (B) in a multi-anvil (MA) device in Dr. D. Walker's lab at Columbia University.

2. Difference between synthesis techniques.

One sample was quenched at 4 GPa in both PC and MA to determine the difference between these two techniques. The MA device yielded greater change in the structure and properties than the PC device at the same nominal pressure, resulting in higher refractive index and higher density increase at 4 GPa (Fig. S2). This may be partially due to quench rate difference between these two devices. In other words, the MA may lock in a higher density with a faster quench rate that the PC with a slower quench rate. Additional source of error for effective pressure experienced by the sample during quenching involves the pressure drop that accompanies rapid cooling. This effect was mitigated with the PC apparatus by adjusting the screw-pump which controls the hydraulic pressure on the piston while the temperature dropped. With this technique, pressure deviation during the quench process was isobaric within ~10% for the 19-mm assembly and ~5% for the 13-mm assembly. The maximum deviation occurred within the first few seconds following power termination, then pressure could be compensated for the remaining quench process. For the MA apparatus, the Aremco 584OF compound has been previously determined to remain < 200°C at the experiment temperature in this work (internal study). Small octahedron volume and insignificant temperature increase of the Aremco compound yield negligible thermal pressure effects due to thermal expansion and subsequent contraction upon power termination. Because of the unspecified effects at the sample due to differences in pressure delivery techniques, and differences in quenching and unloading times, the nominal calibration errors for intended pressure delivery define the error bars in this study. In the rest of our study, all data for the HC-4 GPa sample are from the one synthesized in the PC apparatus.

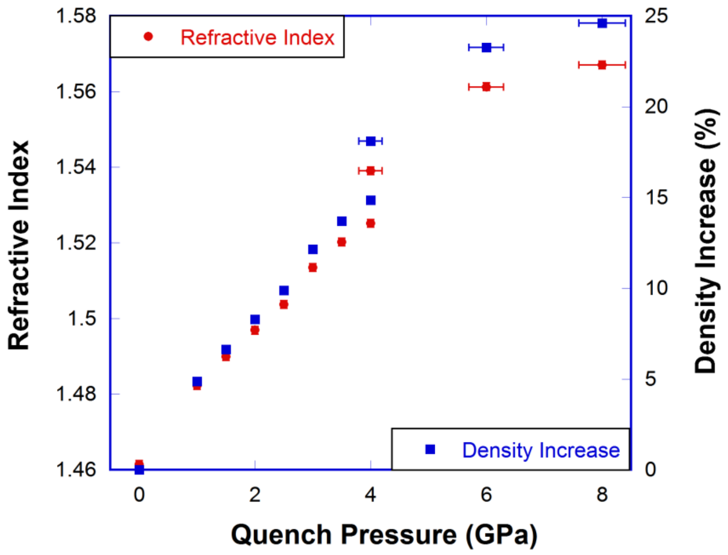

Fig. S2. Refractive index and density increase vs. quench pressure, showing relatively higher density from the MA device compared to the PC device at nominal applied pressure of 4 GPa.

3. Density of amorphous and crystalline silica.

Tan and Arndt conducted an extensive study on many densified silica glass samples; with over 100 samples tested they were able to determine quite well a linear relationship between refractive index and density, with a slope of 0.195 up to 16.5% densification[6]. Density of several crystalline phases of silica also fall onto the linear refractive index vs. density relationship as seen in Fig. S3. This linear relationship was used to calculate the density of hot-compressed silica glass samples in this study.

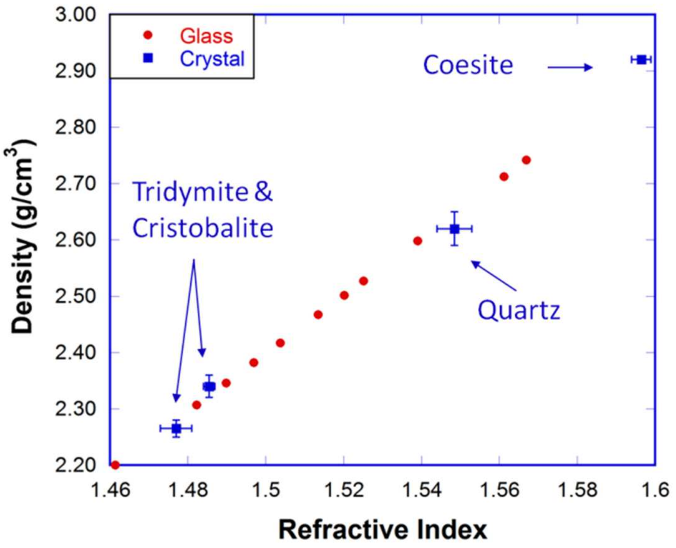

Fig. S3. Density versus refractive index of crystalline silica phases and densified silica glass, all with tetrahedrally coordinated Si.

4. First sharp diffraction peak (FSDP).

Fig. S4 shows the position and the full width at half maximum (FWHM) of the first sharp diffraction peak (FSDP) as a function of quench pressure measured by X-ray diffraction at ambient conditions.

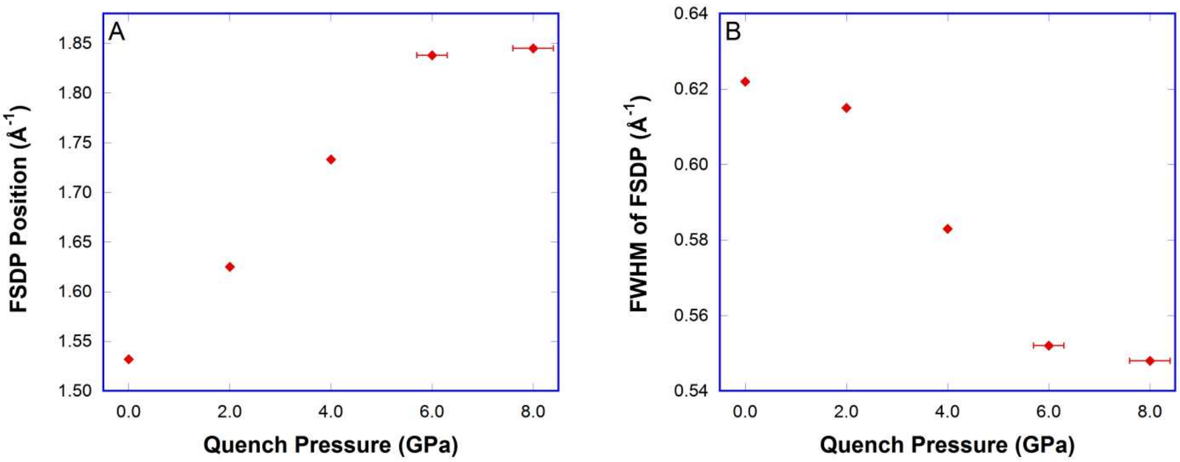

Fig. S4. (A) Position and (B) full width at half maximum (FWHM) of FSDP as a function of quench pressure.

Total X-ray structure factor for silica glass samples hot-compressed in our MD simulation are shown in Fig. S5.

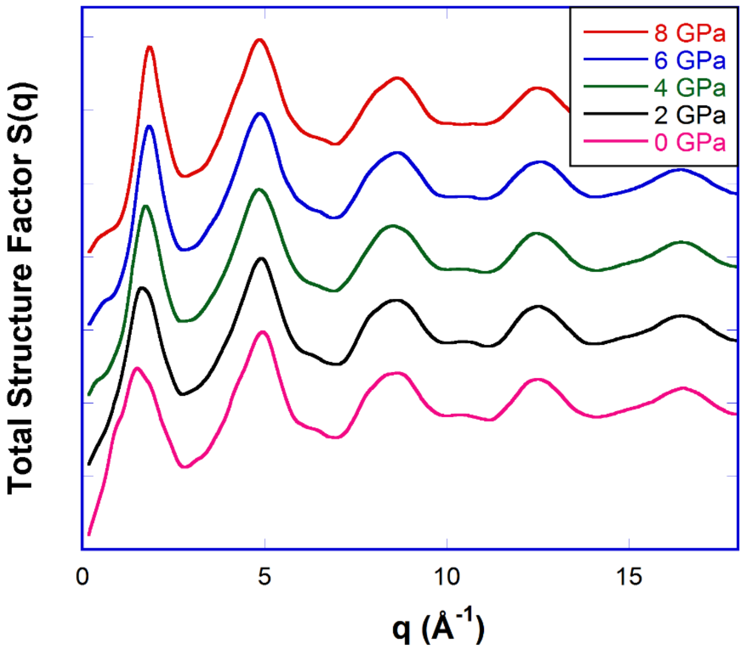

Fig. S5. Calculated total X-ray structure factor for silica glass hot-compressed under 2, 4, 6 and 8 GPa, compared to that of pristine silica glass (0 GPa) in MD simulation.

5. Real space correlation.

Radial distribution function, *G(r)*, is a one-dimensional function that oscillates around one showing positive peaks at distances from a reference atom where the local atomic density exceeds the average (Eq. S1-S2). For the G(r) of pristine silica glass, the following attributions are made: $r_{Si\text{-}O}$=1.59 Å (Fig. S6B), $r_{O\text{-}O}$=2.60 Å (Fig. S6C), $r_{Si\text{-}Si}$=3.07 Å (Fig. S6D), and $r_{Si\text{-}O2}$=4.07 Å (Fig. S6E)[7,8]. The Si-O2 distance is from silicon to the second nearest oxygen, which was postulated initially by Bell and Dean[9] to contribute significantly to the FSDP.

It can be seen from Fig. S6 that all of the first several peaks show some shift in distances with the increase of quench pressure, including Si-O distance from 1.586 Å to 1.595 Å, a 0.57% increase (Fig. S6B). Si-O bond lengthening of 0.43% has also been determined by electron spin resonance of silica glass with 24% density increase[10].

$$G(r) = 4\pi\rho_o\left[\frac{\rho(r)}{\rho_o - 1}\right] \quad \text{(S1)}$$

$$G(r) = \frac{2}{\pi}\int_{q=0}^{q_{\max}} q\left[S(q)-1\right]\sin(qr)dq \quad \text{(S2)}$$

Based on the G(r) of hot-compressed samples, the maximum changes in atomic distances for the sample quenched under 8 GPa are: $\Delta r_{Si-O} = 0.57\%$, $\Delta r_{O-O} = 2.13\%$, $\Delta r_{Si-Si} = 0.39\%$, $\Delta r_{Si-O2} = 0.99\%$. In pristine silica glass, $\theta_{Si-O-Si} = 149.87°$ was calculated based on the Si-O and the Si-Si distances in G(r). For the HC-6 GPa sample, $\theta_{Si-O-Si} = 146.45°$ was obtained. A sharp jump in the Si-Si distance for the HC-8 GPa sample gives $\theta_{Si-O-Si} = 148.16°$. As determined by MD simulation coupled with neutron diffraction, the peaks beyond the first O-O correlation are composite peaks with Si and O contribution[8], perhaps leading to the perceived Si-Si distance increase for the HC-8 GPa sample and the increased Si-O2 distance for the sample series studied here.

In the cold-compression, XRD studies have indicated an initial decrease in Si-O bond distance, then an increased distance with onset pressures of $9 < P < 28$ GPa[11], or $P > 15$ GPa[12]. This has been interpreted as an increase in Si coordination number. At 40-50 GPa the Si-O bond length is close to that of stishovite, an octahedrally coordinated high pressure silica polymorph[11-13]. Interestingly, no silica glass with 6-fold coordinated Si atoms has ever been recovered from high pressure experiments[14,15]. The high temperature synthesis conditions in our study are unlikely to aid higher Si coordination states at lower pressures, since the transition between coesite and stishovite is displaced to higher pressures as temperature is increased[16]. Some silicate glasses of various compositions have been identified to retain 5 and 6-fold silicon atoms upon return from elevated pressures[15,17,18], but ^{29}Si MAS NMR showed no coordination increase in SiO_2 glass which was quenched from liquid at 6 GPa, with a detection limit of 0.3%[15]. By oxygen number density, even our glass of highest density increase (25%) is well within the expected range for tetrahedral coordination[19]. Total Si-O bond distance increase of 0.57% is small enough to rule out significant silicon coordination changes in our hot-compressed samples. Therefore, changes in the short-range order are excluded from contributing to the bulk behaviors of hot-compressed silica glass.

The G(r) intensity is seen to decrease between 5 and 9 Å (Fig. S6A), but increase near 4 Å. Changes near 5 Å are pronounced when silica glass was compacted in the rigid state as reported

by Mukai et al.[20], but here the significant decrease is at 6.25 Å. With increased correlation near 4 Å and less correlation at 5 and 6.25 Å, it seems that the intermediate range order is not diminished but rather shifted to shorter distances in hot-compressed samples.

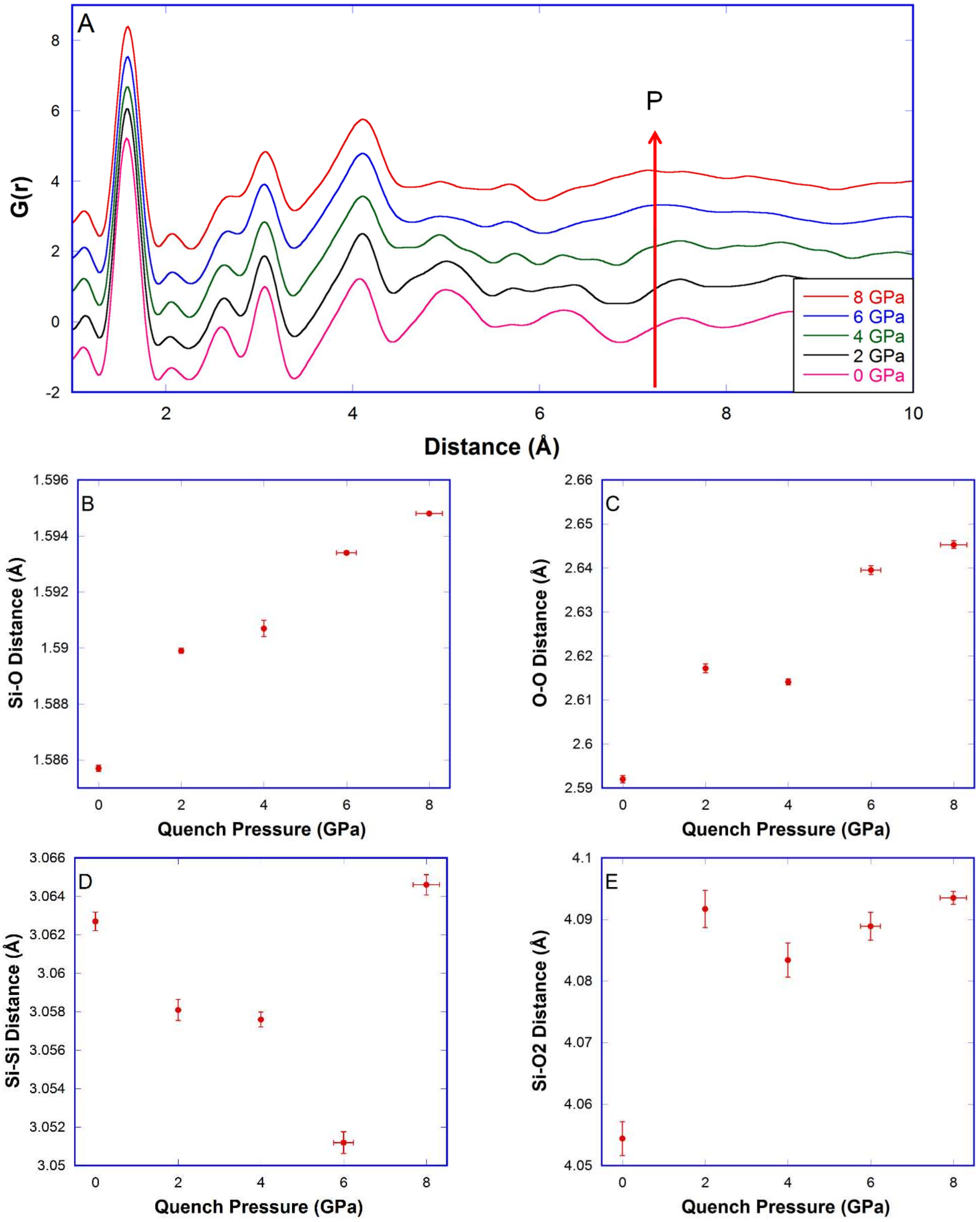

Fig. S6. (A) G(r) spectra for hot-compressed silica glass. (B) Si-O, (C) O-O, (D) Si-Si and (E) Si-O2 (Si to second nearest O) as a function of quench pressure.

6. Raman frequency shift.

With network forces dominating the vibration behaviors, the central force model (CFM) predicts that each tetrahedral mode gives way to a lower and a higher frequency bands. The isolated "A_1" breathing mode of symmetric oxygen motion gives rise to one type of network vibration that is higher than the "F_2" mode of asymmetric oxygen motion. Sarnthein et al. showed by first-principles density functional theory calculations that the high frequency Raman bands are driven by these tetrahedral molecular modes[21]. The 1050 cm^{-1} band is due to the F_2 mode and the 1200 cm^{-1} band results from the A_1 tetrahedral breathing mode, opposite in relative frequency from the isolated tetrahedral modes. However, the idealized tetrahedral motions are themselves lost, modified by the random network connectivity in silica glass. This is exemplified in the 1200 cm^{-1} Raman peak shift to lower frequencies by 10 cm^{-1} when ^{30}Si was substituted for ^{28}Si[22], for which the isolated tetrahedra would show no shift in the A_1 mode with immobile Si at the center. As the Si-O-Si angle decreases, symmetric and antisymmetric contributions converge in frequency, which is evidenced by the main band shifting to higher frequencies (Fig. S7A) and the high frequency bands shifting toward lower values with the increase of quench pressure (Fig. S7B)[23].

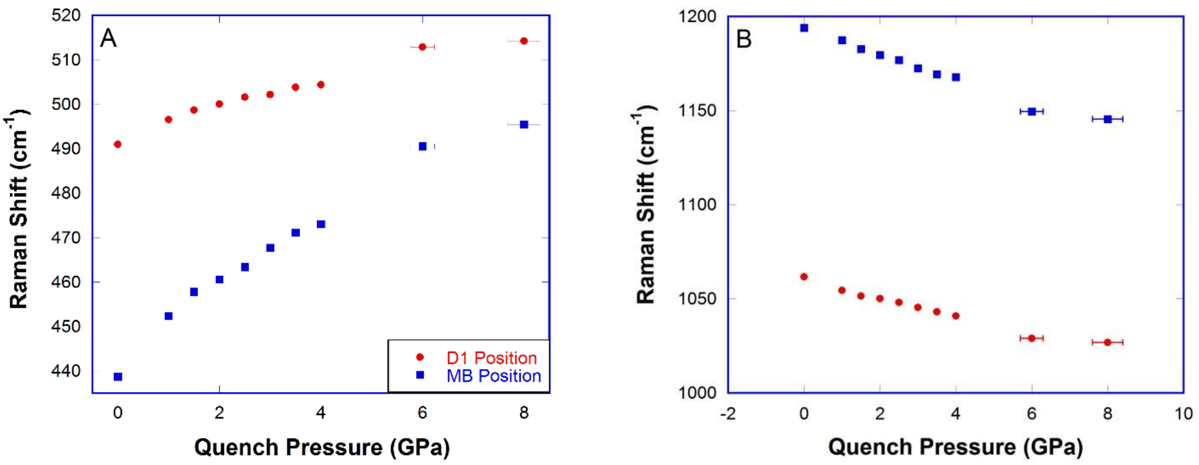

Fig. S7. (A) Shift of the main band (MB) and the D_1 peak and (B) high frequency Raman bands with the increase of quench pressure.

Raman spectra of cold-compressed and hot-compressed silica glass with a similar densification (~14% density increase) are shown in Fig. S8.

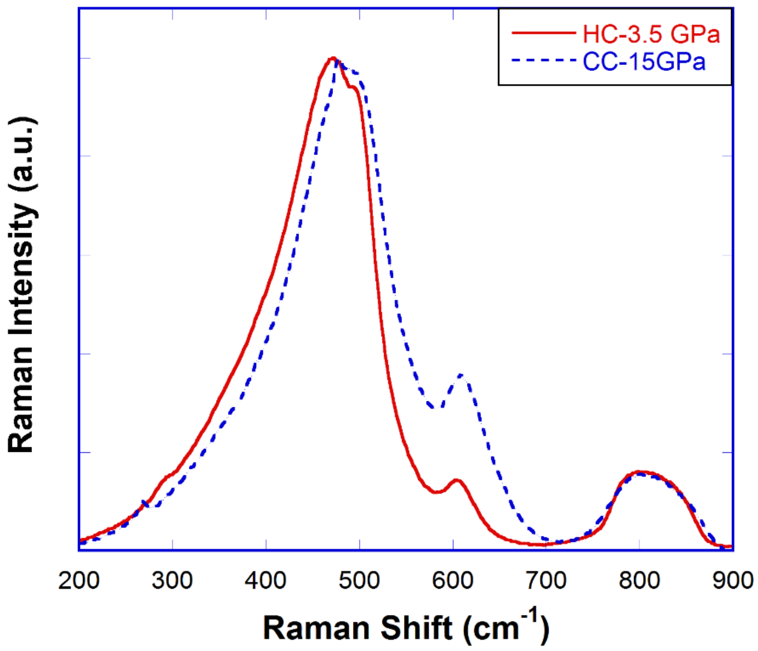

Fig. S8. Raman spectrum of HC-3.5 GPa sample and CC-15 GPa sample (both with density increase of ~14%), measured at ambient conditions.

7. Ambient Elastic moduli.

Brillouin light scattering (BLS) measures the frequency shift of photons scattered by thermal excitations (phonons), which naturally exist in condensed matter at finite temperatures[24] and propagate at a velocity that is characteristic of the material. From the longitudinal (V_L) and transverse (V_T) sound velocities measured by BLS in the 'emulated platelet geometry'[25], the elastic moduli including the Poisson's ratio can be determined:

$$G = V_T^{\,2}\rho \tag{S3}$$

$$C11 = V_L^{\,2}\rho \tag{S4}$$

$$E = V_T^{\,2}\left(\frac{3V_L^{\,2} - 4V_T^{\,2}}{V_L^{\,2} - V_T^{\,2}}\right)\rho \tag{S5}$$

$$K = \left(V_L^{\,2} - \frac{4}{3}V_T^{\,2}\right)\rho \tag{S6}$$

$$\nu = \frac{V_L^2 - 2V_T^2}{2(V_L^2 - V_T^2)} \tag{S7}$$

where G=C_{44} is the shear modulus, C_{11} is the longitudinal modulus, E and K are the Young's modulus and bulk modulus, ν is Poisson's ratio, and ρ is the sample density. As seen in Fig. S9, the Poisson's ratio increases with the density increase in both hot-compressed and cold-compressed samples[26]. A higher maximum value of Poisson's ratio of 0.252 was reported by Rouxel et al. for cold-compressed silica glass with 21.6% density increase[27].

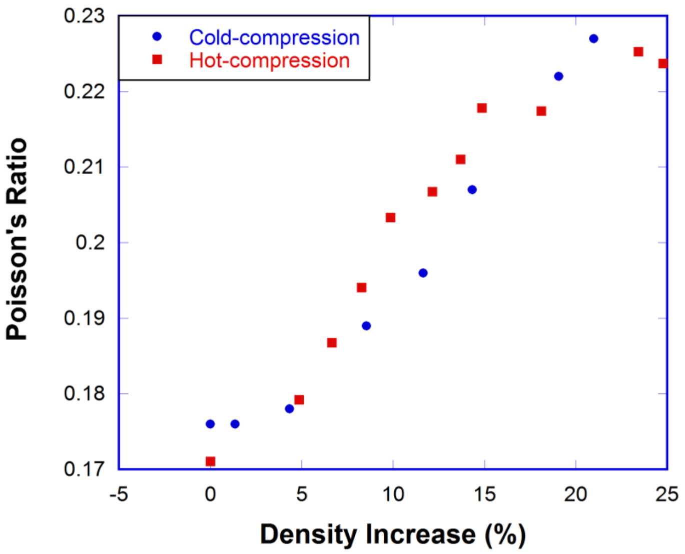

Fig. S9. Poisson's ratio vs. density increase of silica glass from the cold-compression at room temperature[26] and the hot-compression at 1100°C in this work, measurements were done at ambient conditions.

Young's modulus and shear modulus are shown to increase more readily with the density increase up to ~20% (Fig. S10A-B) in the hot-compression than in the cold compression, as also seen for bulk modulus (Fig. 3B).

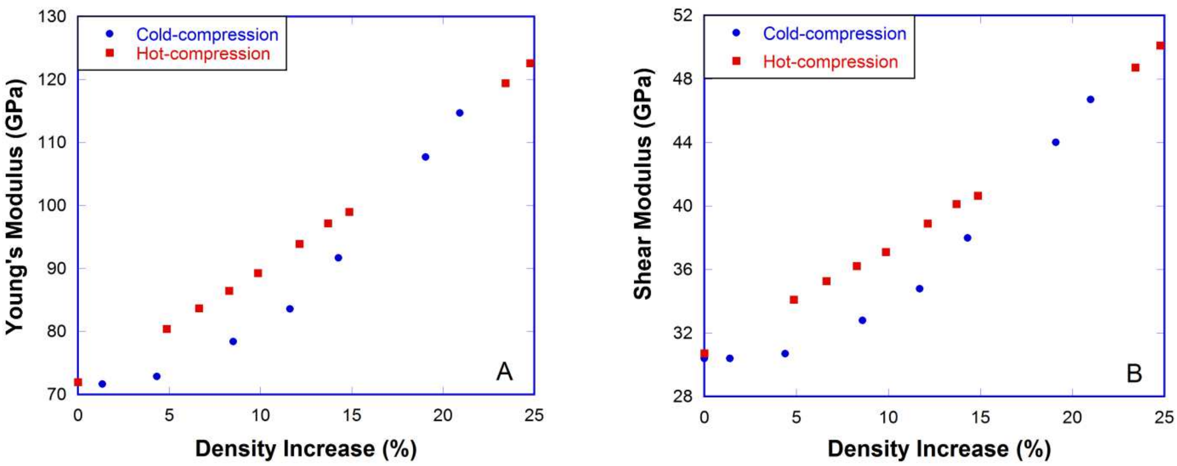

Fig. S10. (A) Young's modulus and (B) shear modulus vs. density increase of silica glass from the cold-compression at room temperature[26] and the hot-compression at 1100°C in this work, all measurements were done at ambient conditions.

8. Elastic response to pressure.

As shown in previous studies, silica glass can be permanently densified when compressed beyond the threshold of 8-9 GPa at ambient temperature[28-31]. This behavior was reproduced in Fig. S11A in our study. During compression, the longitudinal Brillouin frequency shift initially decreases with increasing pressure; it reaches a minimum near 2.5 GPa, and then increases through

15 GPa. The frequency shift during decompression is higher than that during compression over the entire pressure range from 0 to 15 GPa. The frequency shift in the recovered glass at ambient conditions increases by 12.5% with respect to pristine silica glass. The hysteresis during the compression-decompression cycle in pressure-quenched samples gradually diminishes with the increase of quench pressure as seen in Fig. S11B-D. In the HC-4 GPa sample, the minimum in the frequency shift remains noticeable in the compression and decompression paths, but the decompression path is reversible. For the HC-6 and HC-8 GPa samples, both the minimum in frequency shift and the hysteresis loop are completely vanquished, and they deform elastically to at least 20 and 26 GPa, respectively, which were the highest pressures tested.

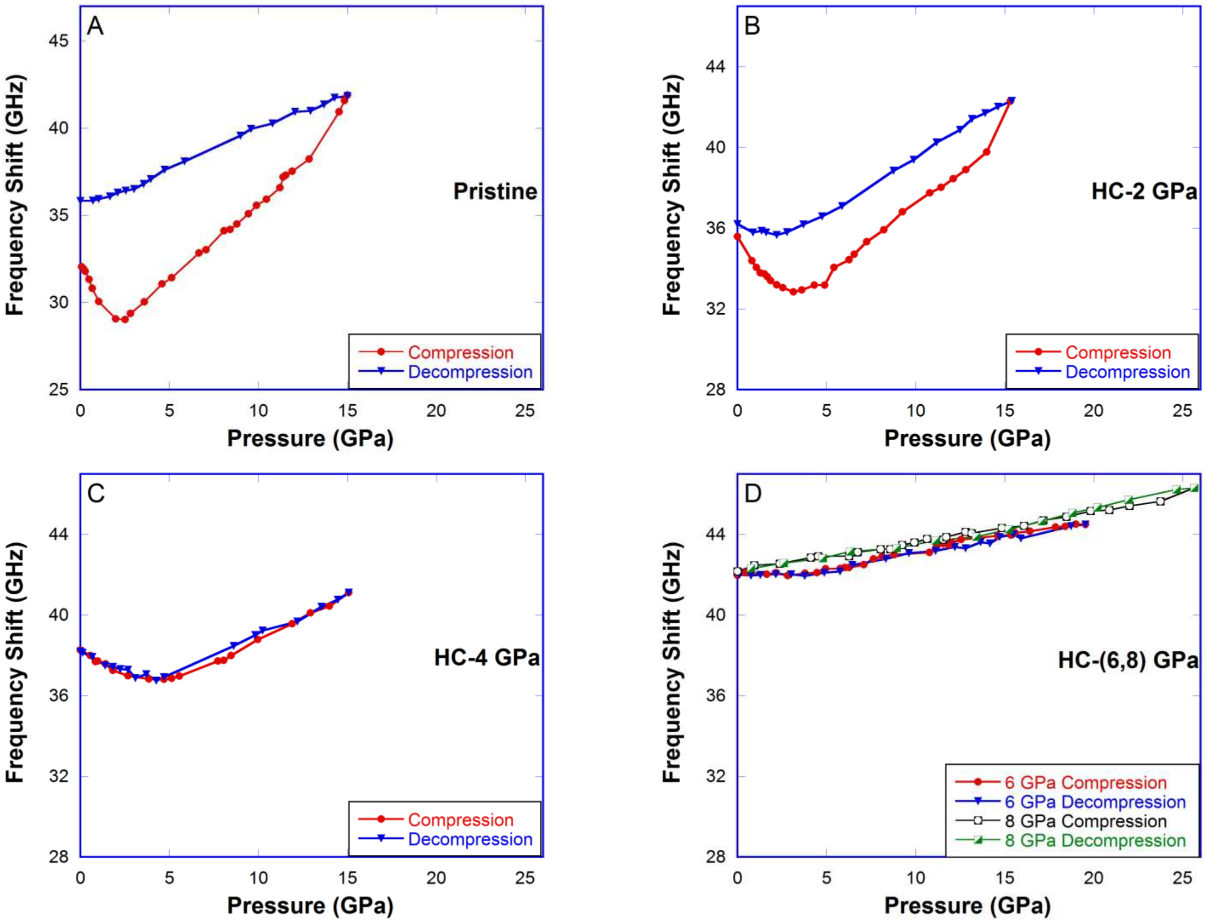

Fig. S11. Longitudinal Brillouin frequency shift as a function of pressure during compression and decompression cycle in (A) pristine, (B) HC-2 GPa, (C) HC-4 GPa and (D) HC-6 and HC-8 GPa samples. Note: in (A)-(C), all samples were tested in methanol-ethanol mixture as the pressure transmitting medium, in (D), samples were tested in Ar.

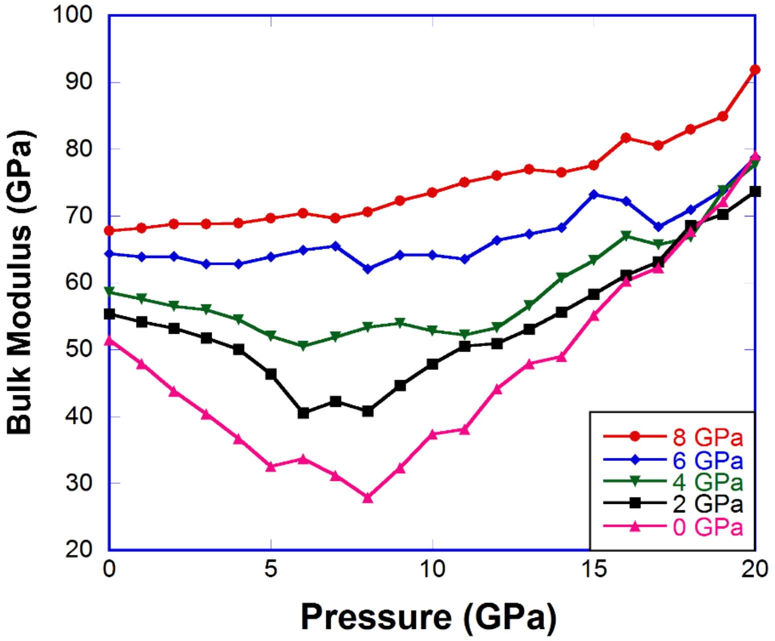

Fig. S12. Bulk modulus of hot-compressed silica glass as a function of testing pressure during compression up to 20 GPa at room temperature from MD simulation.

9. Fitting notes.

FSDP positions in Fig. S4A were determined by normalizing and isolating the peak from 0.5 to 1, fitting the peak to an asymmetric Gaussian function[32]. For the asymmetric Gaussian, a smoothly varying full width at half maximum (FWHM) function was used as described by Stancik and Brauns for fitting asymmetric infrared absorption spectra[32]. Fitting errors are within the symbol size. FWHM values in Fig. S4B were visually determined by magnifying the normalized spectra at 0.5 intensity, with 0.003 $Å^{-1}$ per pixel resolution for errors within symbol size.

Si-O distances in Fig. S6B were determined by isolating normalized peak intensity from 0.5 to 1 and using a single Gaussian fit. O-O and Si-Si distances near 2.6 and 3.1 Å were fit with a double Gaussian function after a linear background subtraction between the minima near 2.2 and 3.4 Å. The Si-O2 peak near 4.1 Å was fit with an asymmetric Gaussian function[32] after a linear background subtraction between the minima near 3.4 and 4.5 Å. Error bars reflect the given fitting errors.

The normalized Raman spectra were isolated from 0.5 to 1 to reduce asymmetry, then the MB-D1 double peak was fit with an asymmetric Gaussian to describe the MB, and a symmetric Gaussian to describe D1 peak. MB and D1 peak positions were resolved from the doublet by this technique, and are represented in Fig. S7A. Error bars reflect the given fitting errors, which are within the symbol size. High frequency Raman peak positions in Fig. S7B were fit with Gaussian (~1060 cm^{-1}) and asymmetric Gaussian (~1200 cm^{-1}) functions. Fitting errors are also within the symbol size.

References

1. Watson, E., Wark, D., Price, J. & Orman, J. V. Mapping the thermal structure of solid-media pressure assemblies. *Contr. Mineral. Petrol.* **142**, 640-652 (2002).
2. Walker, D. Lubrication, Gasketing, and Precision in Multianvil Experiments. *Am. Mineral.* **76**, 1092-1100 (1991).

3. van Westrenen, W., Van Orman, J. A., Watson, H., Fei, Y. & Watson, E. B. Assessment of temperature gradients in multianvil assemblies using spinel layer growth kinetics. *Geochem Geophys* **4**, 1036 (2003).
4. Huang, L. & Kieffer, J. Anomalous thermomechanical properties and laser-induced densification of vitreous silica. *Appl. Phys. Lett.* **89**, 141915 (2006).
5. Li, C.-Y. *Ph.D. Thesis, Rensselaer Polytechnic Institute*, (2012).
6. Tan, C. Z., Arndt, J. & Xie, H. S. Optical properties of densified silica glasses. *Phys. B Condens. Matter* **252**, 28-33 (1998).
7. Lucovsky, G. & Phillips, J. C. Nano-regime Length Scales Extracted from the First Sharp Diffraction Peak in Non-crystalline SiO_2 and Related Materials: Device Applications. *Nanoscale Res. Lett.* **5**, 550-558 (2010).
8. Susman, S. *et al.* Intermediate-Range Order in Permanently Densified Vitreous SiO_2 - a Neutron-Diffraction and Molecular-Dynamics Study. *Phys. Rev. B* **43**, 1194-1197 (1991).
9. Bell, R. J. & Dean, P. The structure of vitreous silica: Validity of the random network theory. *Philos. Mag.* **25**, 1381-1398 (1972).
10. Devine, R. A. B. & Arndt, J. Si—O bond-length modification in pressure-densified amorphous SiO_2. *Phys. Rev. B* **35**, 9376-9379 (1987).
11. Meade, C., Hemley, R. J. & Mao, H. K. High-pressure x-ray diffraction of SiO_2 glass. *Phys. Rev. Lett.* **69**, 1387-1390 (1992).
12. Benmore, C. J. *et al.* Structural and topological changes in silica glass at pressure. *Phys. Rev. B* **81**, 054105 (2010).
13. Sato, T. & Funamori, N. Sixfold-Coordinated Amorphous Polymorph of SiO_2 under High Pressure. *Phys. Rev. Lett.* **101**, 255502 (2008).
14. Williams, Q. & Jeanloz, R. Spectroscopic evidence for pressure-induced coordination changes in silicate glasses and melts. *Science* **239**, 902-905 (1988).
15. Xue, X. Y., Stebbins, J. F., Kanzaki, M., McMillan, P. F. & Poe, B. Pressure-Induced Silicon Coordination and Tetrahedral Structural-Changes. *Am. Mineral.* **76**, 8-26 (1991).
16. Swamy, V., Saxena, S. K., Sundman, B. & Zhang, J. A thermodynamic assessment of silica phase diagram. *J. Geophys. Res.: Solid Earth* **99**, 11787-11794 (1994).
17. Stebbins, J. F. NMR evidence for five-coordinated silicon in a silicate glass at atmospheric pressure. *Nature* **351**, 638-639 (1991).
18. Stebbins, J. F. & McMillan, P. 5- and 6-coordinated Si in $K_2Si_4O_9$ Glass Quenched from 1.9 GPa and 1200°C. *Am. Mineral.* **74**, 965-968 (1989).
19. Zeidler, A., Salmon, P. S. & Skinner, L. B. Packing and the structural transformations in liquid and amorphous oxides from ambient to extreme conditions. *Proc. Natl. Acad. Sci* **111**, 10045-10048 (2014).
20. Rahmani, A., Benoit, M. & Benoit, C. Signature of small rings in the Raman spectra of normal and compressed amorphous silica: A combined classical and ab initio study. *Phys. Rev. B* **68**, 184202 (2003).
21. Sarnthein, J., Pasquarello, A. & Car, R. Origin of the high-frequency doublet in the vibrational spectrum of vitreous SiO_2. *Science* **275**, 1925-1927 (1997).
22. Galeener, F. L. & Geissberger, A. E. Vibrational dynamics in 30Si-substituted vitreous SiO_2. *Phys. Rev. B* **27**, 6199-6204 (1983).
23. McMillan, P., Piriou, B. & Couty, R. A Raman-Study of Pressure-Densified Vitreous Silica. *J. Chem. Phys.* **81**, 4234-4236 (1984).
24. Sandercock, J. R. Trends in Brillouin scattering: Studies of opaque materials, supported films, and central modes. *Top. Appl. Phys.* **51**, 173-206 (1982).

25. Guerette, M. & Huang, L. A simple and convenient set-up for high-temperature Brillouin light scattering. *J. Phys. D: Appl. Phys.* **45**, 275302 (2012).
26. Deschamps, T., Margueritat, J., Martinet, C., Mermet, A. & Champagnon, B. Elastic Moduli of Permanently Densified Silica Glasses. *Sci. Rep.* **4**, 7193 (2014).
27. Rouxel, T., Ji, H., Guin, J. P., Augereau, F. & Rufflé, B. Indentation deformation mechanism in glass: Densification versus shear flow. *J. Appl. Phys.* **107**, 094903 (2010).
28. Grimsditch, M. Polymorphism in amorphous SiO_2. *Phys. Rev. Lett.* **52**, 2379-2381 (1984).
29. Polian, A. & Grimsditch, M. Room-temperature densification of a-SiO_2 versus pressure. *Phys. Rev. B* **41**, 6086-6087 (1990).
30. Rouxel, T., Ji, H., Hammouda, T. & Moreac, A. Poisson's ratio and the densification of glass under high pressure. *Phys. Rev. Lett.* **100**, 225501 (2008).
31. Sonneville, C. *et al.* Progressive transformations of silica glass upon densification. *J. Chem. Phys.* **137**, 124505 (2012).
32. Stancik, A. L. & Brauns, E. B. A simple asymmetric lineshape for fitting infrared absorption spectra. *Vib. Spectrosc.* **47**, 66-69 (2008).
